# The Hidden Variable in Radiological Accuracy: The Impact of Monitor Quality Under Real-Life Emergency Department Conditions

**DOI:** 10.3390/tomography12030043

**Published:** 2026-03-20

**Authors:** Bahadir Caglar, Suha Serin

**Affiliations:** Department of Emergency Medicine, Faculty of Medicine, Balikesir University, 10900 Balikesir, Turkey; suhaserin@gmail.com

**Keywords:** diagnostic imaging, radiological displays, emergency department

## Abstract

Background/Objectives: Radiological assessment has become indispensable for modern clinical decision-making. Image quality plays a critical role in the reliability of radiological interpretation. Unlike most previous studies, this study investigated the effect of monitor type on diagnostic accuracy and ease of diagnosis under physical conditions outside the radiology unit. Methods: Three image sets were prepared for the study, consisting of emergency radiological images, each containing 50 computed tomography, magnetic resonance imaging, and digital radiography images. The image sets were examined by five emergency specialists, who were blinded to each other’s work, under emergency service conditions on a standard monitor (SM), medical monitor (MM), and advanced monitor (AM). The accuracy and ease of diagnosis were analyzed statistically according to the type of monitor used. Results: Overall diagnostic accuracy rates were 98.7% for SM, 100% for AM, and 100% for MM. Cochran’s Q test demonstrated a statistically significant difference between monitor types (*p* = 0.002), with significant pairwise differences for SM–AM and SM–MM comparisons. The absolute risk difference between SM and AM/MM was 1.3%, corresponding to a relative risk of 1.013 and a number needed to benefit (NNB) of 77. Ease of diagnosis scores increased progressively across monitor types (SM: 7.6 [IQR 7–8], AM: 9.4 [IQR 9–9.8], MM: 9.8 [IQR 9.6–10]; *p* < 0.001), with a large overall effect size (Kendall’s W = 0.81). Multilevel modeling confirmed that these associations persisted after adjustment for clustering effects. Conclusions: In situations where medical monitors cannot be used due to cost and operational constraints, opting for advanced monitors instead of standard monitors may modestly improve diagnostic accuracy while substantially enhancing perceived ease of diagnosis.

## Simple Summary

Radiological images are increasingly interpreted outside radiology reading rooms, particularly in emergency departments, where medical monitors may not always be available. Although medical displays are considered the reference standard, advanced monitors are becoming more accessible. This study evaluated whether monitor type influences diagnostic accuracy and perceived ease of diagnosis under real-life emergency department conditions. Although differences in diagnostic accuracy were statistically significant, the absolute improvement was modest. However, advanced monitors significantly improved perceived ease of diagnosis and demonstrated diagnostic accuracy comparable to medical monitors. These findings suggest that advanced monitors may represent a practical alternative to standard monitors in high-volume emergency care settings.

## 1. Introduction

Radiological imaging has become an essential component of modern patient management and clinical decision-making [1]. With the widespread implementation of Picture Archiving and Communication Systems (PACSs), access to radiological images is no longer confined to the radiology department. Consequently, non-radiologist physicians, particularly in acute care settings, are increasingly required to interpret images for immediate clinical management.

The reliability of these evaluations depends heavily on image quality. Therefore, the gold standard for image interpretation in radiology units involves the use of medical monitors. These monitors strictly adhere to the Digital Imaging and Communications in Medicine (DICOM) standards, specifically the Grayscale Standard Display Function (GSDF) based on the Barten Model [2]. This calibration ensures perceptual linearity, meaning that the monitor provides accurate grayscale representation and consistent luminance across the entire brightness range, which is essential for detecting subtle pathologies.

However, outside of dedicated radiology reading rooms, the adoption of medical monitors is limited by their high acquisition costs. In resource-constrained environments such as emergency departments (EDs), intensive care units (ICUs), and outpatient clinics, radiological images are routinely evaluated on standard off-the-shelf monitors. Unlike medical displays, these standard monitors often lack routine DICOM calibration and fail to meet optimal luminance requirements. This discrepancy raises concerns regarding potential diagnostic errors, especially in the high-pressure, high-volume environment of the ED.

Technological advancements have recently made high-resolution consumer-grade monitors offering increased contrast ratios and higher brightness more accessible and cost-effective compared to traditional medical displays. While the literature suggests that high luminance monitors (minimum 450 cd/m^2^) may offer advantages in diagnostic accuracy, the clinical impact of substituting medical monitors with high-end consumer monitors remains underinvestigated [3,4]. Furthermore, previous studies assessing monitor performance have predominantly been conducted under idealized conditions with minimal ambient light (0 lux) [3,5]. Such conditions do not reflect the ecological reality of EDs, which are characterized by uncontrolled ambient lighting, noise, and varying viewing angles that can degrade grayscale perception.

To address this gap in the literature, this study aims to assess the impact of three different display types on radiological diagnostic accuracy and interpretive ease under real-world emergency department conditions with standard ambient lighting.

## 2. Materials and Methods

This study was supported by a scientific research project at Balikesir University, Balikesir, Turkey (Project No: 2022/003). The Balikesir University Ethics Committee granted local ethics committee approval for the study (Approval Date and No: 19 April 2023/2023-44). The study was conducted in the emergency department of Balikesir University Hospital between 2023 and 2025.

### 2.1. Image Sets and Monitors

A blinded radiologist team (consisting of three radiology specialists) prepared image sets containing 50 computed tomography (CT), magnetic resonance imaging (MRI), and digital radiography (DR) images for the study. The image sets were derived from emergency radiological cases to reflect the real-world clinical environment under investigation. Because the primary aim of the study was to evaluate monitor performance under emergency department conditions, case selection was limited to imaging studies commonly encountered in acute care settings. Cases were selected to represent a range of clinically relevant pathologies typically requiring prompt interpretation. None of the users participating in the study had seen these cases before. The radiologist who prepared the cases selected them specifically from cases that the emergency department had previously consulted them about. Image evaluations were conducted in three separate areas within the same emergency department room under routine overhead lighting conditions. Ambient illumination was approximately 500 lux, based on the standard lighting specifications of the clinical area. Lighting conditions remained unchanged throughout all evaluation sessions. Participants were seated at a consistent viewing distance, and monitor positioning was kept stable across sessions. Reflections and glare were not quantitatively measured. Desktop tower PCs of the same make and model (Dell Vostro) connected to PACS were installed in all areas. The computers were equipped with a standard monitor (SM) (Dell P2219H, Round Rock, TX, USA, 1920 × 1080, 250 cd/m^2^, 1000:1; USD 90) in the first area, an advanced monitor (AM) (Dell P2423D, Round Rock, TX, USA, 2560 × 1440, 300 cd/m^2^, 1000:1; USD 268) in the second area, and a 3MP medical monitor (MM) (Eizo RadiForce GX340, Hakusan, Japan, 1536 × 2048, 1200 cd/m^2^, 1400:1; USD 1500) in the third area.

The medical monitor was factory calibrated in accordance with the DICOM Grayscale Standard Display Function (GSDF) and included built-in luminance stabilization. The advanced and standard monitors were used under manufacturer default display settings without formal DICOM GSDF calibration, reflecting routine emergency department practice. Objective luminance uniformity measurements were not independently performed during the study period.

### 2.2. Users

Five emergency medicine specialists with 10–15 years of professional experience were selected as users. All participating physicians had self-reported normal or corrected-to-normal vision. None reported known visual impairment affecting image interpretation. Participants were allowed to use their habitual corrective lenses during evaluation. Users were asked to evaluate the image sets on three different monitors at 1-week intervals. A 1-week washout interval was implemented between evaluation sessions to minimize potential recall bias related to repeated image assessment. Similar washout periods have been used in observer performance and ROC-based imaging studies to reduce memory effects while preserving study feasibility [6,7]. Before each evaluation, the images within the image sets were shuffled. This was done to prevent users from adapting to the images. A balanced crossover rotation scheme was applied to distribute monitor exposure across users and image sets in a counterbalanced manner (Table 1). The user numbers of the physicians were determined by lottery.

### 2.3. Application

The image sets were accessed via PACS. While evaluating the images, the use of zoom, magnification, and contrast adjustment features was permitted. No fixed time limit was imposed during image evaluation. This approach was intentionally chosen to avoid introducing artificial time pressure that could confound assessment of monitor-related performance. Participants interpreted images at their usual clinical pace to reflect routine emergency department practice. During the study, the physicians evaluating the images were kept blind to each other’s work. Users were not provided with demographic or clinical information about the cases. For each image, the monitor type, diagnostic accuracy, and ease of diagnosis were recorded. Diagnoses were compared with those of the radiology specialist. Following evaluation of each image set on each monitor type, participants provided a single global subjective rating using a 10-point Likert scale (1 = very difficult, 10 = very easy). In the manuscript and tables, this measure is referred to as “ease of diagnosis.” Operationally, this rating reflected the participants’ overall perceived ease of establishing a diagnosis, incorporating both interpretive ease and subjective comfort during image evaluation. This study-specific, single-item measure was designed to capture overall perceived interpretive experience rather than discrete constructs such as diagnostic confidence, visual fatigue, or cognitive workload.

### 2.4. Statistical Analysis

Data analysis was performed using IBM SPSS Statistics for Windows, Version 25.0 (IBM Corp., Armonk, NY, USA) software.

#### 2.4.1. Sample Size and Power Analysis

An a priori sample size calculation was performed using GPower (v3.1.9.7, Heinrich-Heine-University Düsseldorf, Germany). Given the repeated measures design in which the same images were evaluated across three monitor types (SM, AM, MM), diagnostic accuracy (binary outcome) was treated as paired data. Because Cochran’s Q test is not directly implemented in GPower, a conservative McNemar-based approach was used for primary pairwise comparisons.

A clinically meaningful absolute accuracy difference of 7.5% (within a 5–10% anticipated range) was assumed for planning purposes. With Bonferroni-adjusted α = 0.025 and 80% power, the minimum required sample size was estimated at 127 images. To enable balanced modality-specific analyses (50 DR, 50 CT, 50 MRI), the final sample size was set at 150 images.

#### 2.4.2. Descriptive Statistics

Continuous variables were expressed as medians (Q1–Q3), and categorical variables as n (%). Statistical significance was defined as *p* < 0.05 (two-sided).

#### 2.4.3. Diagnostic Accuracy Analysis

Overall differences in diagnostic accuracy across monitor types were assessed using Cochran’s Q test (Monte Carlo method). When significant, pairwise comparisons were conducted using McNemar’s test with Bonferroni correction.

Effect size measures were additionally calculated to facilitate clinical interpretation, including absolute risk difference (RD), relative risk (RR), continuity-corrected odds ratio (OR), and number needed to benefit (NNB).

Interobserver agreement for diagnostic accuracy was assessed using Fleiss’ Kappa with 95% confidence intervals, interpreted according to Landis and Koch (1977). To account for potential prevalence effects, prevalence-adjusted bias-adjusted Kappa (PABAK) values were also calculated using the formula PABAK = 2P_o_ − 1, where P_o_ represents observed agreement. This adjustment is recommended in datasets with highly imbalanced category distributions, where conventional Kappa statistics may underestimate true agreement due to inflated expected agreement values. Both Fleiss’ Kappa and PABAK values were reported to provide a more comprehensive evaluation of interobserver agreement.

#### 2.4.4. Ease of Diagnosis Analysis

Ease of diagnosis scores (1–10 Likert scale) were analyzed using the Friedman test (Monte Carlo method) due to non-normal distribution. Effect size for overall comparison was reported as Kendall’s W. Post hoc pairwise comparisons were performed with Bonferroni correction.

Inter-user reliability was assessed using a two-way mixed effects model with absolute agreement and average measures [ICC (3, 5)], interpreted according to Koo and Li (2016).

#### 2.4.5. Generalized Linear Mixed Models (GLMMs)

To account for repeated measurements and a cross-classified data structure (multiple readers evaluating multiple images), generalized linear mixed models (GLMMs) were constructed. For diagnostic accuracy (binary outcome), a binomial distribution with logit link function was used. For ease of diagnosis (continuous outcome), a linear mixed effects model with identity link was applied.

Image and reader were included as cross-classified random intercepts. Monitor type and image set were included as fixed effects, with SM and DR as reference categories. Model-based estimated marginal means (EMMEANS) were used for pairwise comparisons, with Bonferroni correction.

Model fit was evaluated using −2 log likelihood, Akaike information criterion (AIC), and Bayesian information criterion (BIC). Marginal R^2^ (fixed effects only) and conditional R^2^ (fixed + random effects) were calculated. Intraclass correlation coefficients (ICCs) were derived from variance components. All tests were two-sided, with *p* < 0.05 considered statistically significant.

#### 2.4.6. Majority Vote Approach

Because the primary aim of the study was to compare monitor performance, a majority vote approach was also applied. For each image, the responses of the five readers were aggregated to generate a consensus classification.

For example, if at least three of the five readers correctly identified digital radiography image number 2 on the SM according to the reference standard, the case was classified as a true vote. Conversely, if at least three readers provided an incorrect interpretation, the case was classified as a “false vote” (Table 2).

This approach was intended to reduce the influence of individual reader variability and provide a monitor-level comparison based on collective diagnostic decisions.

## 3. Results

### 3.1. Results of Analyses Conducted for Diagnostic Accuracy

Overall diagnostic accuracy and inter-observer agreement were high across all monitor types; however, statistically significant differences were observed between monitors (Table 2).

#### 3.1.1. Diagnostic Accuracy Analysis Results in the Digital Radiography (DR) Image Set

The diagnostic accuracy rate was found to be 98% with standard monitor (SM) use, 100% with advanced monitor (AM) use, and 100% with medical monitor (MM) use. Cochran’s Q test showed that the difference among SM, AM, and MM was statistically significant (*p* < 0.001). In the post hoc analysis, the difference remained significant in both the SM–AM and SM–MM pairs (*p* < 0.001), while no difference was observed between AM and MM (*p* > 0.05). Cross tables based on users show that true/true cells are predominant (over 90%) among users 1, 2 and 3 in particular, and that the rates of misdiagnosis (false/false or discordant) did not exceed 2–4% (Table 3). These findings indicate high diagnostic consistency among users in the DR set and a significant increase in the correct diagnosis rate as monitor resolution increases. In the effect size analysis, Cramér’s V values range from 0.105 to 0.275 on a user basis, varying between small and medium levels. This indicates that, while the difference is statistically significant, the effect size is limited. The Fleiss Kappa values are SM = 0.072, AM = −0.033, and MM = −0.020, indicating insignificant agreement according to the Koo and Li classification. However, the prevalence- and bias-adjusted Kappa (PABAK) values were calculated as 0.96 for SM and 1.00 for AM and MM, indicating that inter-observer agreement is in fact nearly perfect. This finding supports the notion that low Kappa values stem not from actual disagreement, but from statistical limitations associated with high true diagnosis prevalence.

#### 3.1.2. Diagnostic Accuracy Analysis Results in Computed Tomography (CT) Image Sets

The correct diagnosis rates in the CT set were 100%, 100%, and 100% on the SM, AM, and MM, respectively. No significant difference was observed at the global level (*p* > 0.05). However, when examined on a user basis, a significant difference was found for user 5 (*p* = 0.024), suggesting that individual variation is more dependent on the observer than on the monitor type. Cramér’s V values range from 0.000 to 0.209, mostly indicating a small effect size. Although Fleiss Kappa values (SM = −0.053; AM = 0.020; MM = −0.029) appear low, the fact that PABAK values are calculated as 1.00 for all monitors indicates that the observed agreement is excellent. Therefore, the low Kappa coefficients in the CT set do not indicate a true lack of agreement, but are related to the homogeneity of the category distribution.

#### 3.1.3. Diagnostic Accuracy Analysis Results in the Magnetic Resonance Imaging (MRI) Image Set

Accuracy rates in the MRI set were found to be 98% for SM and 100% for AM and MM. Cochran’s Q test did not show a significant difference at the global level (*p* > 0.05), but significant differences were found for user 2 (*p* = 0.028) and user 5 (*p* = 0.008) at the user level. Cross tables show that the “true/true” rate reached 96–98% among users, particularly on the MM. Cramér’s V values range from 0.083 to 0.214, indicating a small to moderate effect, and suggest that monitor type has a limited but measurable impact for some users. Fleiss Kappa values are SM = 0.020, AM = 0.026, and MM = −0.050, which are low; however, PABAK values were calculated as 0.96 for SM and 1.00 for AM and MM, confirming that inter-observer agreement is actually high. This situation is consistent with the Kappa paradox, which is associated with an increase in expected agreement under conditions of high prevalence.

#### 3.1.4. Overall Evaluation (All Modalities)

In the total analysis, where findings from three different image sets were combined into a single image set, the overall accuracy rate was found to be SM = 98.7%, AM = 100%, and MM = 100%. Cochran’s Q test showed a significant difference among monitors (*p* = 0.002). In the post hoc test, the SM–AM (*p* < 0.001) and SM–MM (*p* < 0.001) comparisons were significant; there was no difference between AM and MM (*p* > 0.05). In the overall analysis, the absolute risk difference between SM and AM/MM was 1.3%. The relative risk was 1.013. The number needed to benefit (NNB) was calculated as 77. Cramér’s V values ranged from 0.051 to 0.209, indicating small to moderate effect sizes and suggesting that statistical significance reflects effects of limited magnitude. Fleiss Kappa values were calculated as SM = 0.019, AM = 0.010, and MM = −0.033, while PABAK values were found to be 0.97, 1.00, and 1.00, respectively, revealing that inter-observer agreement was practically perfect.

As monitor quality increased, a statistically significant but small to moderate effect size increase in diagnostic accuracy was observed. Cramér’s V results indicate that the difference is measurable but limited. In contrast, raw agreement rates and PABAK values reveal nearly perfect consistency among observers across all monitor types. When these findings are considered together, it is understood that the low Fleiss Kappa values reflect a statistical limitation due to high category prevalence rather than a true lack of agreement.

In evaluations conducted on three different monitors, both the advanced user monitor and the medical monitor provided statistically higher diagnostic accuracy compared to the standard monitor; however, effect size analyses indicate that this difference is small to moderate. Although inter-observer agreement appears low according to the Kappa coefficient, PABAK and raw agreement rates reveal that all users produced parallel results within a 95–100% accuracy range. Therefore, it was concluded that monitor quality has a measurable but limited effect on diagnostic performance, particularly in DR and MRI images, while monitor type is not a determining factor in CT images.

### 3.2. Results from Analyses Conducted for Ease of Diagnosis

Ease of Diagnosis showed a significant difference across all imaging modalities (digital radiography, CT, and MRI) depending on monitor type (*p* < 0.001, Friedman test—Monte Carlo). When all modalities were evaluated together, the global effect size was calculated as Kendall’s W = 0.810, which corresponds to a very large effect level and demonstrates that the monitor type has a strong and clinically meaningful effect on diagnostic ease.

Median (Q1–Q3) values showed a steady increase from the SM to the AM and MM for both individual users and user averages. This finding indicates that high-resolution monitors (especially medical monitors) significantly increase ease of diagnosis (Table 4).

#### 3.2.1. Digital Radiography (DR) Image Set Ease of Diagnosis Analysis Results

In the digital radiography group, the effect size for user averages was found to be Kendall’s W = 0.750, and this value was assessed as large to very large. In individual user analyses, effect sizes ranged from 0.472 to 0.599, indicating that the effect of monitor type on diagnostic ease was moderate to large. The average ease of diagnosis scores were SM = 8.1 (7.6–8.4), AM = 9.2 (9–9.4), and MM = 9.8 (9.2–10). Both SM→AM and SM→MM comparisons were statistically significant (*p* < 0.001), but the AM↔MM difference was not significant (*p* = 0.073). This indicates that even advanced monitors provide a significant advantage over standard monitors in terms of diagnostic comfort, but medical monitors only increase this gain to a limited extent. In terms of inter-observer consistency, almost perfect agreement was found, with ICC (3, 5) = 0.869 (0.802–0.919). This indicates that there is a high level of consensus among users and that the results are reliable.

#### 3.2.2. Computed Tomography (CT) Image Set: Ease of Diagnosis Analysis Results

Ease of diagnosis scores in CT images also increased significantly with monitor resolution (*p* < 0.001). The Kendall’s W value for user averages was calculated as 0.868, which corresponds to a very large effect size. Effect sizes in individual user analyses ranged from 0.774 to 0.902, all of which are at the large to very large effect size level. The mean scores were SM = 7.6 (7–8), AM = 9.4 (9–9.8), and MM = 9.8 (9.8–10). The differences between SM→AM and SM→MM were statistically significant (*p* < 0.001), while the difference between AM↔MM was minimal (*p* = 0.006). This result confirms that monitor resolution increases diagnostic comfort, particularly in high-contrast and detail-demanding images such as CT. Inter-observer consistency was excellent, with ICC = 0.872 (0.804–0.921) for SM and ICC = 0.857 (0.784–0.912) for AM. The low ICC in MM (ICC = 0.170) is likely due to the ceiling effect (where every user gives the maximum score).

#### 3.2.3. Magnetic Resonance Imaging (MRI) Image Set Ease of Diagnosis Analysis Results

In the MRI analysis, the global effect size was calculated as Kendall’s W = 0.823, which corresponds to a very large effect size. Individual user effect sizes ranged from 0.812 to 0.857, all falling into the very large effect size category. Diagnostic ease in MRI was lowest in SM (7.2 (6.6–7.8)) and highest in MM (10 (9.6–10)). The difference was highly significant in both SM→AM and SM→MM comparisons (*p* < 0.001). In magnetic resonance images, monitor quality is thought to significantly improve perceptual clarity, particularly due to high contrast richness. Inter-observer reliability is quite high in MRI sets (ICC= 0.924 SM, 0.921 AM, 0.849 MM), indicating a high level of agreement among users in their assessments.

#### 3.2.4. Overall Evaluation (All Modalities)

Findings from three different image sets were combined into a single image set for total analysis; overall diagnostic ease scores were determined as SM = 7.6 (7–8), AM = 9.4 (9–9.8), and MM = 9.8 (9.6–10) (*p* < 0.001). Both the SM→AM and SM→MM differences were statistically significant (*p* < 0.001), while the AM↔MM difference was partially significant (*p* ≤ 0.05). This finding indicates that advanced monitors significantly improved perceived diagnostic ease, while medical monitors provided optimal diagnostic comfort. Inter-user reliability is excellent, with ICC (3, 5) = 0.879 (0.846–0.907). These results confirm that observer variability is minimal and consistency is high across different monitor types.

The findings reveal that monitor type has a direct effect on diagnostic comfort. Even advanced monitors provided a clear advantage over standard monitors, but medical monitors offered the optimum level of diagnostic ease. High ICC values among observers support that these findings are not subjective, but rather repeatable and reliable.

#### 3.2.5. Multilevel Modeling Analysis (GLMM)

To account for the cross-classified data structure (multiple readers evaluating multiple images), generalized linear mixed models (GLMMs) were constructed, with image and reader included as random intercepts.

Diagnostic accuracy: A GLMM with binomial distribution and logit link function was used. SM was defined as the reference category. Compared to SM, AM was associated with significantly higher diagnostic accuracy (β = 1.38, SE = 0.30, OR = 3.99, 95% CI: 2.20–7.25, *p* < 0.001). Similarly, MM demonstrated higher diagnostic accuracy relative to SM (β = 1.50, SE = 0.32, OR = 4.49, 95% CI: 2.41–8.34, *p* < 0.001). No statistically significant difference was observed between AM and MM (β = −0.12, SE = 0.38, OR = 0.89, 95% CI: 0.42–1.89, Bonferroni-adjusted *p* = 0.999) (Table 5).

Model-based marginal probabilities were estimated at 94% for SM, 98% for AM, and 99% for MM. These model-adjusted probabilities differ slightly from raw observed percentages due to adjustment for clustering at reader and image levels. The relatively large odds ratios reflect the low baseline error rate, where small absolute differences may correspond to amplified relative effect estimates.

Image set effects: Using DR as the reference category, no significant difference was observed between CT and DR (*p* = 0.465). However, MRI images were associated with significantly lower diagnostic accuracy compared to DR (β = −0.74, SE = 0.29, OR = 0.48, 95% CI: 0.27–0.83, *p* = 0.009). No significant difference was observed between MRI and CT (*p* = 0.139).

Ease of diagnosis: A linear mixed effects model with identity link function was applied for the continuous ease of diagnosis scores. SM was used as the reference category. Compared to SM, AM was associated with significantly higher scores (β = 1.75, SE = 0.04, *p* < 0.001), and MM showed an even greater increase (β = 2.18, SE = 0.04, *p* < 0.001). Additionally, MM was significantly associated with higher scores compared to AM (β = 0.43, SE = 0.04, *p* < 0.001), indicating a graded relationship (SM < AM < MM). Model-based mean scores were 7.54 for SM, 9.30 for AM, and 9.72 for MM.

With respect to image set effects for ease of diagnosis, no significant difference was observed between CT and DR (*p* = 0.097), whereas MRI was associated with significantly lower scores compared to DR (β = −0.14, SE = 0.06, *p* = 0.015). No difference was found between MRI and CT (*p* = 0.721).

Random effects and model fit: In the diagnostic accuracy model, clustering was observed at both image (ICC = 10.4%) and reader (ICC = 14.2%) levels. In the ease of diagnosis model, clustering was modest at the image level (ICC = 10.4%) and negligible at the reader level (ICC = 0.5%). For diagnostic accuracy, marginal R^2^ was 0.11 and conditional R^2^ was 0.33, indicating meaningful contribution of random effects. For ease of diagnosis, marginal R^2^ was 0.64 and conditional R^2^ was 0.68, suggesting that fixed effects accounted for most of the explained variance. Model fit was evaluated using −2 log likelihood, AIC, and BIC. All *p* values were reported after Bonferroni correction.

## 4. Discussion

Emergency departments are environments characterized by heavy workloads, high stress, and the need for rapid decision-making under multitasking conditions. Under these conditions, physicians’ visual perception load increases, the risk of error rises, and radiological diagnosis becomes critical [8].

In this context, understanding how display characteristics influence diagnostic performance becomes particularly important in emergency department practice.

This study examined the effects of monitor types on diagnostic accuracy and ease of diagnosis in the evaluation of DR, CT, and MRI data for emergency radiological diagnoses.

Our findings indicate that monitor type may influence diagnostic performance, particularly in terms of perceived ease of diagnosis. According to current guidelines, radiological images should ideally be evaluated on medical monitors [2,9,10]. In the present study, advanced monitors demonstrated diagnostic accuracy comparable to medical monitors, although medical displays provided the highest level of diagnostic comfort during image interpretation.

Although a statistically significant difference in diagnostic accuracy was observed between monitor types, the absolute difference was modest (1.3%). Effect size analysis demonstrated a relative risk of 1.013 and a number needed to benefit (NNB) of 77, indicating that approximately one additional correct diagnosis would be expected for every 77 image evaluations when using advanced or medical monitors instead of a standard monitor. Although the odds ratios appear relatively large, this is largely attributable to the very low baseline error rate. In high accuracy settings, small absolute differences can yield inflated relative effect estimates. Therefore, absolute risk difference and NNB may provide more clinically interpretable measures than relative statistics alone.

These findings underscore the distinction between statistical and clinical significance. While the multilevel analysis confirmed that the association persisted after adjustment for reader and image-level clustering, the practical relevance of a 1–2% improvement depends largely on clinical context. Although this magnitude of effect may appear small at the individual case level, its impact may become more meaningful in high-volume emergency department settings. For example, in a centre interpreting approximately 1000 radiological examinations per year, a 1.3% increase in diagnostic accuracy may correspond to approximately 13 additional correct diagnoses.

Importantly, the present study evaluated not only diagnostic accuracy but also perceived ease of diagnosis. Ease of diagnosis scores demonstrated a large overall effect size (Kendall’s W = 0.81), indicating that monitor quality substantially influenced the interpretive experience of clinicians. Although parameters such as decision-making time, cognitive workload, and reporting efficiency were not directly measured in this study, improved interpretive ease may plausibly contribute to these clinically relevant aspects of diagnostic workflow.

From a practical perspective, advanced monitors may represent a feasible alternative in settings where medical displays are not available. However, decisions regarding display investment should be guided by institutional workload, case complexity, acceptable diagnostic risk thresholds, and cost–benefit considerations rather than statistical significance alone.

The uniformly high diagnostic accuracy observed in CT images may reflect the inclusion of clearly diagnosable emergency pathologies, potentially contributing to a ceiling effect. This may partly explain why monitor-related differences were less pronounced in CT compared to DR and MRI.

Across monitor types, observed agreement (P_o_) ranged between 96% and 100%, indicating near-perfect concordance among readers. Under such high-prevalence conditions, the expected chance agreement (Pe) becomes mathematically inflated, which may attenuate Fleiss’ Kappa values despite high raw agreement. Therefore, the near-zero or negative Kappa coefficients observed in this study reflect a prevalence-related statistical limitation rather than true interobserver disagreement. This phenomenon, often referred to as the “Kappa paradox,” is well described in high-prevalence datasets and does not indicate methodological error.

In emergency department environments where standard monitors are used, clinicians may experience greater difficulty during image interpretation, which could potentially contribute to increased diagnostic uncertainty or additional consultation requests.

However, all monitor studies in the literature have been conducted under darkroom conditions in radiology units. To our knowledge, no previous studies have evaluated monitor performance under standard room lighting conditions typical of emergency departments. As our study was conducted under emergency department conditions, the data obtained reflect real-life situations.

### 4.1. Technical Requirements and Advantages of Medical Monitors

Medical displays are devices specifically manufactured for diagnostic evaluation, featuring technological infrastructure that ensures compliance with the DICOM standard, high contrast ratio, automatic brightness calibration, and homogeneous illumination. The literature reports that medical screens are superior to standard monitors in terms of grayscale accuracy and contrast ratio: while standard monitors have an average contrast ratio of 500:1, that for medical displays can reach 2000:1. Furthermore, thanks to Grayscale Standard Display Function (GSDF) compliance, a more accurate visual response is obtained in detecting low-contrast differences [2].

These technical advantages are particularly relevant in cases involving subtle findings or low-contrast abnormalities, such as small nodules or microcalcifications. In a comparison between medical displays and commercial displays, medical displays were found to be significantly superior [9].

### 4.2. The Prevalence of Standard Monitors, Reasons for Their Use, and Potential Risks

Under routine clinical conditions, the use of medical monitors in emergency departments is not always feasible. Because of cost, maintenance requirements, and budget limitations, standard monitors are more commonly used in everyday practice. However, compared with medical displays, their lower brightness, contrast, and resolution may reduce grayscale fidelity and increase the risk that low-contrast lesions are overlooked. Therefore, although standard monitors are more accessible, their use may pose a potential limitation in terms of diagnostic accuracy and patient safety.

### 4.3. Resolution, Monitor Type, and Evaluation Performance

Studies evaluating the relationship between monitor resolution and clinical decision-making from different perspectives exist in the literature. It has been reported that 12 MP monitors reduce assessment time by an average of 6–7 s compared to 6 MP monitors [10,11]. This finding suggests that monitor performance may indirectly contribute to workflow speed in high-paced environments such as emergency departments.

However, no significant difference was found between 3 MP medical monitors and 1 MP standard monitors in mammography images [12]. Another study observed that low resolution had a limited effect on diagnosis [13].

### 4.4. Cost–Benefit Balance and Real-World Implementation Challenges

The cost of medical monitors is an important consideration in institutional investment decisions. Cost–benefit assessment should include not only acquisition cost, but also calibration, maintenance, technical service, replacement frequency, and service life. Previous reports have noted monitor malfunctions, brightness decline, and replacement needs over time [14]. Accordingly, although standard monitors may offer a more economical option, the decision to adopt medical displays should be guided by the clinical setting, case complexity, and acceptable diagnostic risk.

### 4.5. Ease of Use

Our study evaluated “ease of diagnosis” as a global subjective assessment of the interpretive experience, a parameter that has not been explicitly examined in previous display comparison studies. In this study, ease of diagnosis was measured using a single-item rating reflecting the overall perceived ease of establishing a diagnosis, incorporating both interpretive ease and subjective comfort during image evaluation. Importantly, this measure was not designed to directly quantify diagnostic confidence, cognitive workload, or visual fatigue as independent constructs.

Conceptually, perceived ease of diagnosis may relate to cognitive load theory and principles of perceptual ergonomics, as display characteristics can influence subjective interpretive experience even when objective diagnostic accuracy remains comparable. Although these factors were not directly measured, such frameworks provide context for understanding how different display environments may shape clinicians’ interpretive experience.

In our findings, advanced monitors demonstrated significantly higher ease of diagnosis ratings compared to standard monitors, approaching, but not fully reaching, the diagnostic comfort provided by medical displays. In high-demand clinical settings such as emergency departments—where workload, stress, and multitasking are common—improvements in perceived interpretive ease may contribute to a more favorable working experience. While the present study does not establish a direct causal relationship between subjective ease and diagnostic performance, the results suggest that display characteristics may influence clinicians’ overall interpretive experience in a potentially meaningful way. However, because this measure was based on a single study-specific Likert scale, it should be interpreted cautiously as a global subjective indicator.

### 4.6. Limitations and Future Directions

This study has several limitations. First, the “ease of diagnosis” rating represents a study-specific, single-item subjective measure and does not constitute a formally validated psychometric construct. Therefore, it should be interpreted as a global indicator of perceived interpretive experience rather than a comprehensive measure of diagnostic confidence, cognitive workload, or visual fatigue. As such, it does not allow differentiation among specific components such as diagnostic confidence, cognitive workload, or visual fatigue, nor does it permit assessment of internal consistency. While the scale provides a pragmatic global assessment of perceived interpretive experience, future studies incorporating multidimensional validated instruments would enable more comprehensive evaluation of cognitive and ergonomic factors.

All image evaluations were performed by emergency medicine specialists from a single institution, without inclusion of a radiologist comparison group. Radiologists, owing to their specialized training and heightened sensitivity to subtle grayscale variations, may respond differently to display characteristics. In addition, differences in clinical training and diagnostic workflows across medical specialties may influence how display characteristics affect image interpretation. Emergency physicians often interpret images in time-pressured clinical environments, whereas radiologists typically evaluate images under optimized reading conditions. Therefore, the present findings primarily reflect emergency department practice and should not be directly extrapolated to formal radiology reading environments. Future studies including radiologists and readers from multiple specialties and institutions would help improve the external validity and generalizability of these findings.

Environmental conditions were intended to reflect routine emergency department practice; evaluations were conducted in the same clinical room under consistent overhead lighting conditions (approximately 500 lux). However, precise lux measurements, glare quantification, luminance reflections, and standardized viewing angle assessments were not formally performed. Additionally, stress levels, multitasking demands, user fatigue, and eye strain were not directly measured. Although the absence of time constraints was intended to avoid artificial performance bias, it may have limited strict simulation of real-world emergency workflow variability.

From a technical standpoint, although the medical monitor was factory calibrated in accordance with the DICOM Grayscale Standard Display Function (GSDF), formal DICOM calibration and objective luminance uniformity measurements were not performed for the non-medical monitors. Since grayscale standardization and luminance consistency may influence contrast perception, this represents a technical limitation. Furthermore, other display-related variables—such as prior usage duration and potential screen brightness degradation—were not controlled. Finally, the study was conducted in a single center and included physicians from a single specialty, which may limit generalizability. Future multicenter studies involving diverse specialties, standardized calibration protocols, and larger image datasets are warranted.

As radiological image interpretation increasingly occurs outside dedicated radiology reading rooms, understanding the practical implications of display technology has become increasingly relevant in daily clinical practice. Taken together, the present findings contribute to the limited literature on radiological display performance under real-world clinical conditions. Unlike many previous studies conducted in controlled reading-room environments, our study evaluated monitor performance under routine emergency department conditions, including ambient lighting and non-radiologist image interpretation. These findings therefore provide additional insight into how display technology may influence diagnostic interpretation outside dedicated radiology workstations.

## 5. Conclusions

Although medical monitors remain the reference standard in radiologic display technology, their cost and operational requirements may limit widespread implementation outside dedicated radiology reading rooms. In many emergency and critical care settings, standard office monitors are more commonly used due to budgetary and logistical constraints.

In this study conducted under real-life emergency department conditions, advanced monitors demonstrated diagnostic accuracy comparable to medical displays and superior to standard monitors. However, medical monitors provided the highest level of ease of diagnosis during image interpretation. While the absolute improvement in diagnostic accuracy was modest, advanced monitors significantly enhanced perceived diagnostic ease compared with standard monitors.

These findings suggest that advanced monitors may represent a practical and cost-conscious alternative to standard monitors in high-volume emergency settings where full medical implementation is not feasible. However, institutional decisions should consider clinical context, acceptable diagnostic risk thresholds, and cost–benefit balance.

*Take-home message*: Under real-life emergency department conditions, advanced monitors achieved diagnostic accuracy comparable to medical displays and superior to standard monitors, supporting their consideration for clinical image interpretation outside dedicated radiology reading rooms.

## Figures and Tables

**Table 1:** Study overview.

Week	Image Set	User 1	User 2	User 3	User 4	User 5
Week 1	CT	SM	MM	AM	SM	AM
Week 2	DR	AM	SM	MM	MM	SM
Week 3	MRI	MM	AM	SM	AM	MM
Week 4	CT	MM	AM	SM	AM	SM
Week 5	DR	SM	MM	AM	SM	MM
Week 6	MRI	AM	SM	MM	MM	AM
Week 7	CT	AM	SM	MM	MM	MM
Week 8	DR	MM	AM	SM	AM	AM
Week 9	MRI	SM	MM	AM	SM	SM

CT: computed tomography; DR: digital radiography; MRI: magnetic resonance imaging; SM: standard monitor; MM: medical monitor; AM: advanced monitor.

**Table 2:** Comparison of diagnostic accuracy according to monitor type.

	SM		AM		MM		Effect Size (Cramér’s V)	*p*	*p* Value of Pairwise Comparisons		
	False n (%)	True n (%)	False n (%)	True n (%)	False n (%)	True n (%)			SM vs. AM	SM vs. MM	AM vs. MM
Digital Radiography											
VOTE of Users-Diagnosis	1 (2)	49 (98)	0 (0)	50 (100)	0 (0)	50 (100)	0.116	0.999 ^θ^	ns.	ns.	ns.
User 1-Diagnosis	1 (2)	49 (98)	4 (8)	46 (92)	2 (4)	48 (96)	0.118	0.468 ^θ^	ns.	ns.	ns.
User 2-Diagnosis	2 (4)	48 (96)	1 (2)	49 (98)	0 (0)	50 (100)	0.117	0.776 ^θ^	ns.	ns.	ns.
User 3-Diagnosis	5 (10)	45 (90)	2 (4)	48 (96)	1 (2)	49 (98)	0.151	0.138 ^θ^	ns.	ns.	ns.
User 4-Diagnosis	7 (14)	43 (86)	0 (0)	50 (100)	1 (2)	49 (98)	0.275	0.009 ^θ^	0.007	0.028	0.999
User 5-Diagnosis	3 (6)	47 (94)	1 (2)	49 (98)	1 (2)	49 (98)	0.105	0.340 ^θ^	ns.	ns.	ns.
kappa (95% CI)	0.072 (−0.016/0.160)		−0.033 (−0.121/0.055)		−0.020 (−0.108/0.067)						
*p* value for users	0.107 ^k^		0.460 ^k^		0.648 ^k^						
PABAK	0.96		1.00		1.00						
CT											
VOTE of Users-Diagnosis	0 (0)	50 (100)	0 (0)	50 (100)	0 (0)	50 (100)	0.000	0.999 ^θ^	ns.	ns.	ns.
User 1-Diagnosis	4 (8)	46 (92)	3 (6)	47 (94)	2 (4)	48 (96)	0.069	0.853 ^θ^	ns.	ns.	ns.
User 2-Diagnosis	4 (8)	46 (92)	2 (4)	48 (96)	2 (4)	48 (96)	0.084	0.619 ^θ^	ns.	ns.	ns.
User 3-Diagnosis	5 (10)	45 (90)	2 (4)	48 (96)	1 (2)	49 (98)	0.151	0.242 ^θ^	ns.	ns.	ns.
User 4-Diagnosis	3 (6)	47 (94)	1 (2)	49 (98)	1 (2)	49 (98)	0.105	0.625 ^θ^	ns.	ns.	ns.
User 5-Diagnosis	7 (14)	43 (86)	1 (2)	49 (98)	1 (2)	49 (98)	0.209	0.024 ^θ^	0.043	0.043	0.999
kappa (95% CI)	−0.053 (−0.141/0.034)		0.020 (−0.067/0.108)		−0.029 (−0.116/0.059)						
*p* value for users	0.232 ^k^		0.650 ^k^		0.519 ^k^						
PABAK	1.00		1.00		1.00						
MRI											
VOTE of Users-Diagnosis	1 (2)	49 (98)	0 (0)	50 (100)	0 (0)	50 (100)	0.116	0.999 ^θ^	ns.	ns.	ns.
User 1-Diagnosis	6 (12)	44 (88)	4 (8)	46 (92)	3 (6)	47 (94)	0.094	0.515 ^θ^	ns.	ns.	ns.
User 2-Diagnosis	8 (16)	42 (84)	3 (6)	47 (94)	2 (4)	48 (96)	0.180	0.028 ^θ^	0.091	0.028	0.999
User 3-Diagnosis	5 (10)	45 (90)	2 (4)	48 (96)	3 (6)	47 (94)	0.083	0.368 ^θ^	ns.	ns.	ns.
User 4-Diagnosis	7 (14)	43 (86)	2 (4)	48 (96)	3 (6)	47 (94)	0.128	0.265 ^θ^	ns.	ns.	ns.
User 5-Diagnosis	9 (18)	41 (82)	2 (4)	48 (96)	1 (2)	49 (98)	0.214	0.008 ^θ^	0.029	0.009	0.999
kappa (95% CI)	0.020 (−0.068/0.108)		0.026 (−0.061/0.114)		−0.050 (−0.138/0.037)						
*p* value for users	0.656 ^k^		0.557 ^k^		0.260 ^k^						
PABAK	0.96		1.00		1.00						
Total											
VOTE of Users-Diagnosis	2 (1.3)	148 (98.7)	0 (0)	150 (100)	0 (0)	150 (100)	0.094	0.331 ^θ^	ns.	ns.	ns.
User 1-Diagnosis	11 (7.3)	139 (92.7)	11 (7.3)	139 (92.7)	7 (4.7)	143 (95.3)	0.051	0.454 ^θ^	ns.	ns.	ns.
User 2-Diagnosis	14 (9.3)	136 (90.7)	6 (4)	144 (96)	4 (2.7)	146 (97.3)	0.128	0.005 ^θ^	0.043	0.007	0.999
User 3-Diagnosis	15 (10)	135 (90)	6 (4)	144 (96)	5 (3.3)	145 (96.7)	0.128	0.010 ^θ^	0.034	0.015	0.999
User 4-Diagnosis	17 (11.3)	133 (88.7)	3 (2)	147 (98)	5 (3.3)	145 (96.7)	0.180	0.002 ^θ^	0.002	0.01	0.999
User 5-Diagnosis	19 (12.7)	131 (87.3)	4 (2.7)	146 (97.3)	3 (2)	147 (98)	0.209	<0.001 ^θ^	<0.001	<0.001	0.999
kappa (95% CI)	0.019 (−0.032/0.070)		0.010 (−0.40/0.061)		−0.033 (−0.084/0.018)						
*p* value for users	0.461 ^k^		0.687 ^k^		0.200 ^k^						
PABAK	0.97		1.00		1.00						

^θ^ Cochran’s Q test (Monte Carlo), post hoc test: stepwise step-down comparisons, ^k^ Fleiss Kappa, vs.: versus, prevalence-adjusted bias-adjusted Kappa (PABAK), ns.: not significant, observed agreement (Po) corresponds to the proportion of correct classifications (true%) presented in the table. In the overall analysis, P_o_ ranged between 0.96 and 1.00 across monitor types.

**Table 3:** User-based crosstabs based on monitor types.

		Digital Radiography		CT		MRI		Total	
		Image Set on Standard Monitor							
		User 1-SM-Diagnosis		User 1-SM-Diagnosis		User 1-SM-Diagnosis		User 1-SM-Diagnosis	
		False	True	False	True	False	True	False	True
User 2-SM-Diagnosis	False	0	2	0	4	1	7	1	13
	True	4	44	3	43	3	39	10	126
User 3-SM-Diagnosis	False	2	3	0	5	0	5	2	13
	True	2	43	3	42	4	41	9	126
		User 2-SM-Diagnosis		User 2-SM-Diagnosis		User 2-SM-Diagnosis		User 2-SM-Diagnosis	
		False	True	False	True	False	True	False	True
User 3-SM-Diagnosis	False	1	4	0	5	1	4	2	13
	True	1	44	4	41	7	38	12	123
		Image Set on Advanced Monitor							
		User 1-AM-Diagnosis		User 1-AM-Diagnosis		User 1-AM-Diagnosis		User 1-AM-Diagnosis	
		False	True	False	True	False	True	False	True
User 2-AM-Diagnosis	False	0	1	0	2	0	3	0	6
	True	4	45	3	45	4	43	11	133
User 3-AM-Diagnosis	False	0	2	0	2	0	2	0	6
	True	4	44	3	45	4	44	11	133
		User 2-AM-Diagnosis		User 2-AM-Diagnosis		User 2-AM-Diagnosis		User 2-AM-Diagnosis	
		False	True	False	True	False	True	False	True
User 3-AM-Diagnosis	False	0	2	0	2	0	2	0	6
	True	1	47	2	46	3	45	6	138
		Image Set on Medical Monitor							
		User 1-MM-Diagnosis		User 1-MM-Diagnosis		User 1-MM-Diagnosis		User 1-MM-Diagnosis	
		False	True	False	True	False	True	False	True
User 2-MM-Diagnosis	False	0	0	0	2	0	2	0	4
	True	2	48	2	46	3	45	7	139
User 3-MM-Diagnosis	False	0	1	0	1	0	3	0	5
	True	2	47	2	47	3	44	7	138
		User 2-MM-Diagnosis		User 2-MM-Diagnosis		User 2-MM-Diagnosis		User 2-MM-Diagnosis	
		False	True	False	True	False	True	False	True
User 3-MM-Diagnosis	False	0	1	0	1	0	3	0	5
	True	0	49	2	47	2	45	4	141

**Table 4:** Comparison of ease of diagnosis scores according to monitor types.

	SM	AM	MM	Effect Size (Kendall’s W)	*p*	*p* Value of Pairwise Comparisons		
	Median (Q1–Q3)	Median (Q1–Q3)	Median (Q1–Q3)			SM vs. AM	SM vs. MM	AM vs. MM
Digital Radiography								
Means of Users-Ease of Diagnosis	8.1 (7.6–8.4)	9.2 (9–9.4)	9.8 (9.2–10)	0.750	<0.001 ^f^	<0.001	<0.001	0.073
User 1-Ease of Diagnosis	8 (7–8)	10 (8–10)	10 (9–10)	0.537	<0.001 ^f^	<0.001	<0.001	0.329
User 2-Ease of Diagnosis	8 (8–9)	9.5 (9–10)	10 (9–10)	0.563	<0.001 ^f^	<0.001	<0.001	0.363
User 3-Ease of Diagnosis	8 (8–9)	9 (9–10)	10 (9–10)	0.599	<0.001 ^f^	<0.001	<0.001	0.193
User 4-Ease of Diagnosis	8 (8–9)	9 (8–10)	10 (9–10)	0.554	<0.001 ^f^	0.002	<0.001	0.002
User 5-Ease of Diagnosis	8 (8–9)	9 (9–10)	10 (9–10)	0.472	<0.001 ^f^	<0.001	<0.001	0.485
ICC (95% CI)	0.607 (0.405/0.756)	0.023 (−0.483/0.394)	0.869 (0.802/0.919)					
*p* value for users	<0.001	0.441	<0.001					
CT								
Means of Users-Ease of Diagnosis	7.6 (7–8)	9.4 (9–9.8)	9.8 (9.8–10)	0.868	<0.001 ^f^	<0.001	<0.001	0.006
User 1-Ease of Diagnosis	7 (7–8)	9.5 (9–10)	10 (9–10)	0.774	<0.001 ^f^	<0.001	<0.001	0.073
User 2-Ease of Diagnosis	8 (7–8)	9 (9–10)	10 (10–10)	0.823	<0.001 ^f^	<0.001	<0.001	0.073
User 3-Ease of Diagnosis	7 (6–8)	9 (9–10)	10 (10–10)	0.902	<0.001 ^f^	<0.001	<0.001	0.001
User 4-Ease of Diagnosis	8 (7–8)	10 (9–10)	10 (10–10)	0.823	<0.001 ^f^	<0.001	<0.001	0.267
User 5-Ease of Diagnosis	7 (7–8)	9.5 (9–10)	10 (10–10)	0.832	<0.001 ^f^	<0.001	<0.001	0.267
ICC (95% CI)	0.872 (0.804/0.921)	0.857 (0.784/0.912)	0.170 (−0.240/0.479)					
*p* value for users	<0.001	<0.001	0.181					
MRI								
Means of Users-Ease of Diagnosis	7.2 (6.6–7.8)	9.6 (9–10)	10 (9.6–10)	0.823	<0.001 ^f^	<0.001	<0.001	0.193
User 1-Ease of Diagnosis	7 (7–8)	9.5 (9–10)	10 (10–10)	0.812	<0.001 ^f^	<0.001	<0.001	0.999
User 2-Ease of Diagnosis	7 (6–8)	10 (9–10)	10 (9–10)	0.824	<0.001 ^f^	<0.001	<0.001	0.999
User 3-Ease of Diagnosis	7 (7–8)	9 (9–10)	10 (10–10)	0.840	<0.001 ^f^	<0.001	<0.001	0.073
User 4-Ease of Diagnosis	7 (7–8)	10 (9–10)	10 (10–10)	0.857	<0.001 ^f^	<0.001	<0.001	0.531
User 5-Ease of Diagnosis	7 (6–8)	9.5 (9–10)	10 (10–10)	0.833	<0.001 ^f^	<0.001	<0.001	0.329
ICC (95% CI)	0.924 (0.885/0.953)	0.921 (0.875/0.53)	0.849 (0.770/0.906)					
*p* value for users	<0.001	<0.001	<0.001					
Total								
Means of Users-Ease of Diagnosis	7.6 (7–8)	9.4 (9–9.8)	9.8 (9.6–10)	0.810	<0.001 ^f^	<0.001	<0.001	<0.001
User 1-Ease of Diagnosis	8 (7–8)	9 (9–10)	10 (9–10)	0.703	<0.001 ^f^	<0.001	<0.001	0.028
User 2-Ease of Diagnosis	8 (7–8)	9.5 (9–10)	10 (9–10)	0.725	<0.001 ^f^	<0.001	<0.001	0.039
User 3-Ease of Diagnosis	8 (7–8)	9 (9–10)	10 (10–10)	0.774	<0.001 ^f^	<0.001	<0.001	<0.001
User 4-Ease of Diagnosis	8 (7–8)	9 (9–10)	10 (10–10)	0.721	<0.001 ^f^	<0.001	<0.001	<0.001
User 5-Ease of Diagnosis	8 (7–8)	9 (9–10)	10 (9–10)	0.704	<0.001 ^f^	<0.001	<0.001	0.020
ICC (95% CI)	0.879 (0.846/0.907)	0.755 (0.688/0.812)	0.809 (0.756/0.853)					
*p* value for users	<0.001	<0.001	<0.001					

^f^ Friedman test (Monte Carlo); post hoc test: stepwise step-down comparisons, intraclass correlation coefficient (ICC) (model: two-way mixed, absolute agreement (ICC (3, 5)), Q1: 1st Quartile, Q3: 3rd Quartile, vs.: versus.

**Table 5:** GLMM results: effects of monitor and image set on diagnostic accuracy and ease of diagnosis.

		β (SE)	OR	95% CI	Wald z	Adjusted *p* Value
Diagnostic accuracy						
	AM vs. SM	1.38 (0.30)	3.99	2.20–7.25	4.55	<0.001
	MM vs. SM	1.50 (0.32)	4.49	2.41–8.34	4.74	<0.001
	MM vs. AM	−0.12 (0.38)	0.89	0.42–1.89	−0.30	0.999
	Image Set (CT vs. DR)	−0.22 (0.30)	0.8	0.44–1.45	−0.73	0.465
	Image Set (MRI vs. DR)	−0.74 (0.29)	0.48	0.27–0.83	−2.6	0.009
	Image Set (MRI vs. CT)	−0.52 (0.33)	0.6	0.31–1.13	−1.59	0.139
Model Fit Statistics:		AIC: 775.4, BIC: 815.44, marginal R^2^: 0.11, conditional R^2^: 0.33				
Ease of Diagnosis						
	AM vs. SM	1.75 (0.04)	1.75	1.67–1.83	42.61	<0.001
	MM vs. SM	2.18 (0.04)	2.18	2.10–2.26	52.99	<0.001
	MM vs. AM	−0.43 (0.04)	−0.43	−0.51–0.35	−10.38	<0.001
	Image Set (CT vs. DR)	−0.09 (0.06)	−0.09	−0.21–0.02	−1.66	0.097
	Image Set (MRI vs. DR)	−0.14 (0.06)	−0.14	−0.25–0.03	−2.43	0.015
	Image Set (MRI vs. CT)	−0.04 (0.07)	−0.04	−0.18–0.09	−0.64	0.721
Model Fit Statistics:		AIC: 4744.2, BIC: 4790, marginal R^2^: 0.64, conditional R^2^: 0.68				

GLMM (generalized linear mixed model), OR = exp(β); ICC = intraclass correlation coefficient; marginal R^2^ represents the variance explained by fixed effects only; conditional R^2^ represents the variance explained by both fixed and random effects; *p* values were adjusted using the Bonferroni correction.

## Data Availability

Due to privacy concerns, the data presented in this study are available from the corresponding author upon request.
